# Supplementary material for: Resolving the full spectrum of human genome variation using Linked-Reads
Source: Genome Res. 2019 Apr;29(4):635–45. doi: 10.1101/gr.234443.118 (PMC6442396; doi:10.1101/gr.234443.118)
Supplement: Supplemental Material [file supp_gr.234443.118_Supplemental_longranger-2.2.zip › longranger-2.2.tar.bz2!/lr2.2-public/lib/stan/cmdstan/stan/doc/api/html/classstan_1_1mcmc_1_1sample-members.html]

Stan: Member List


|  |  |
| --- | --- |
|  | Stan  2.5.0  probability, sampling & optimization |


- Main Page
- Related Pages
- Namespaces
- Classes
- Files

- Class List
- Class Index
- Class Hierarchy
- Class Members

All Classes Namespaces Files Functions Variables Typedefs Enumerations Enumerator Friends Macros Pages

- stan
- mcmc
- sample

stan::mcmc::sample Member List

This is the complete list of members for stan::mcmc::sample, including all inherited members.

|  |  |  |
| --- | --- | --- |
| accept\_stat() const | stan::mcmc::sample | inline |
| cont\_params(int k) const | stan::mcmc::sample | inline |
| cont\_params(Eigen::VectorXd &x) const | stan::mcmc::sample | inline |
| cont\_params() const | stan::mcmc::sample | inline |
| get\_sample\_param\_names(std::vector< std::string > &names) | stan::mcmc::sample | inline |
| get\_sample\_params(std::vector< double > &values) | stan::mcmc::sample | inline |
| log\_prob() const | stan::mcmc::sample | inline |
| sample(const Eigen::VectorXd &q, double log\_prob, double stat) | stan::mcmc::sample | inline |
| size\_cont() const | stan::mcmc::sample | inline |
| ~sample() | stan::mcmc::sample | inlinevirtual |


---

[ Stan Home Page ]

*© 2011–2014,
Stan Development Team.*
